# Development of Eco-Friendly Polymer Foam Using Overcoat Technology of Deodorant

**DOI:** 10.3390/ma11101898

**Published:** 2018-10-04

**Authors:** Jung Joon Lee, Mi Yeon Cho, Bo-Hyun Kim, Sunjong Lee

**Affiliations:** Research Institute of Sustainable Manufacturing System, Korea Institute of Industrial Technology, Cheonan 31056, Korea; suiya85@naver.com (J.J.L.); aldus1106@kitech.re.kr (M.Y.C.)

**Keywords:** eco-friendly, foam, harmful gas, ammonia, absorption, deodorant

## Abstract

Development of eco-friendly polymer foams is an urgent research topic because of the serious environmental pollution caused by trash heaps and the time-release of harmful gases. Polymer PVC foam using azodicarbonamide as a chemical foaming agent has been highly requested for further improvement due to the residual ammonia gas that continuously leaks out. Here, we demonstrate an effective and costless process for the reduction of releasing ammonia from PVC foams using the overcoat technology of deodorants. We have selected four candidate materials, gelite, zeolite, terra alba, and fumed silica as original materials for the deodorant of ammonia, and they showed an ammonia deodorization rate (ADR) of over 80% without any treatment except the fumed silica. When we over-coated the UV-curing agent mixed deodorants on the PVC foams (thickness ~300 µm), the ADR of the terra alba and zeolite complex foams was remarkably higher than 90%, however, the specific gravity and chromaticity were not changed within 20%. This indicates that our developed process using deodorant layer for ammonia reduction has a high potential for the production of eco-friendly polymer foams.

## 1. Introduction

**Figure 1:**
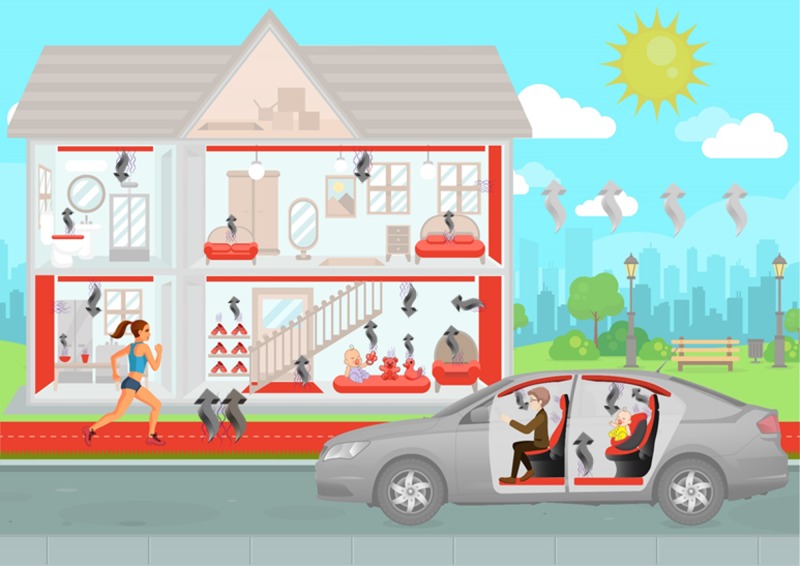
Illustration of the polymer foams usage and the time-releasing gases from the foams in the living environment.

Polymer foams have many useful attributes such as flexibility, low density, impact resistance, and heat transfer capacity compared with conventional rigid polymers [1]. These features make them useful in everyday life for artificial leather, shoes, foam wallpaper, floor coverings, automobile interior trim, mats, and toys and so on as illustrated in Figure 1 [2,3,4,5]. Among the foaming agents that are needed to foam the polymer [6,7,8], azodicarbonamide (ADCA) is the most widely used chemical foaming agent since it has the only self-extinguishing property and excellent storage stability [9,10,11]. However, as the mechanical characteristics of polymer foams are mainly decided by the amount of discharging gases during the thermal decomposing of the forming agent [12,13,14,15], it is inevitable to generate harmful gases such as ammonia and formamide [16]. Although most of the foaming gases are systematically captured by the gas filters, some of them are physically and chemically trapped inside the foam. As time releases the trapped gases from the foams, this can cause chronic health and environmental problems [17,18,19]. Accordingly, the need for reducing or prohibiting the gases from the foams along with the consolidation of world-wide environment regulations has increased and it has become an urgent research target to develop eco-friendly foams [7,20].

So far, various attempts have been made to remove unregulated ammonia, formamide, and formaldehyde leaking from the polymer foams [21,22,23]. For example, a reduction of the releasing gases using additives such as deodorants or metal compounds [20,24,25,26]. However, due to the poor compatibility, the lowering physical properties, and the irregular expansion ratio of the conventional foam, the manufacturing of the uniform characteristic foams is hard using the added deodorant materials. Among the deodorizing materials, a series of pozzolans and fumed silicas have been known to have a good deodorizing ability [27,28,29]. Pozzolan is a natural deodorant that can produce an environmentally friendly foam. However, the deodorant materials mixed with polymer foams were not effective to deodorize the residual gases compared to the original materials [30]. Another method proposed is dehydrogenating the gaseous formamide to hydrogen cyanide (HCN) [31]. However, this method has a disadvantage in the process undertaken at a high temperature (350 to 600 °C) over a long time. Although a modified method was proposed to convert formamide to hydrogen cyanide in a short time (20 s) at a relatively low temperature (220 to 240 °C), it was not economical and was limited by a special thermal surface device coated with a catalyst [32]. Accordingly, an improved method to reduce the releasing gases from the polymer foams must be introduced. Recently, an interesting approach to clay coating on porous foams has been introduced for an alternative fire retardant [33,34], which inspired us to hybridize inorganic deodorants and polymer foams.

In this study, we have probed organic-inorganic hybrid deodorants, which reduce the releasing ammonia gas captured inside the foam. The hybrid deodorants are composed of UV-curable resin and inorganic porous materials such as gelite, zeolite, terra alba, and fumed silica. The porous material’s deodorization rate is preliminarily tested according to the temperature and deodorizing time. The hybrid deodorants are over-coated on the PVC foam body and sequentially UV-cured. The specific gravity and chromaticity of the final foams have been controlled to be changed within 20% compared to the reference. The hybrid deodorants tested the deodorization of ammonia. 

## 2. Experimental

### 2.1. Materials

PVC sol (Dongin Semichem Co. Ltd., Gyeonggi-do, Korea), ADCA chemical foaming agents (Dongin Semichem Co. Ltd.), UV-curing agents (Nano Chemtech Co., Gyeonggi-do, Korea), gelite (Dosung Co. Ltd., Jinju, Korea), zeolite (Kumnong Co., Seongnam-si, Korea), terra alba (Donghae Chemicals Industrial Co. Ltd., Siheung-si, Korea), hydrophilic fumed silica, hydrophobic fumed silica hydrochloric acid (HCl) (35%, Duksan Co. Ltd., Anseong, Korea). Gelite and HCl were mixed by a mechanical stirrer at 100 °C for 1.5 h. Then the HCl-treated gelite was thoroughly washed with water and vacuum dried for 24 h.

### 2.2. Preparation of the Foam

First, PVC sol and 3.4 parts ADCA foaming agent were mixed by a mechanical stirrer. Then the PVC mixture was bar-coated on the release paper (the wet thickness ≈ 1 mm). The bar-coated PVC mixture was foamed in a convection oven at 230 °C for 2 min 10 s [4]. After foaming, the PVC foam was cooled at room temperature for 1 h. A deodorant and UV-curing agent were mixed at a solid ratio 1:1 and dispersed by sonication for 3 min. Then the mixture was bar-coated on the foam. The foam over-coated by the mixture was dried in a convection oven at 40 °C for 15 min and UV-treated for 7 s.

### 2.3. Characterization and Measurements

The surface and cross section morphology of the deodorants and the foams over-coated by the deodorants were measured by scanning electron microscope (SEM) (JEOL/JSM-6701f). The ammonia deodorization rate (ADR) of deodorants was tested by FITI Testing & Research Institute. The specific gravity of the foams over-coated by the deodorants was measured by a hydrometer according to the ASTM-D792 standard test method. The specific gravity of deodorants was calculated from the weight and the volume obtained by using a measuring cylinder. Chromaticity of the foams over-coated by the deodorants was measured by a spectroeye according to the ASTM-E1164 standard test method. Ammonia emission concentration of the foams over-coated by the deodorants was tested by Korea Polymer Testing & Research Institute. The brief test process is: The foams were cut into 2 pieces of 10 cm × 10 cm, respectively. Then each 2 pieces of the cut foams were put in a 5 L Tedlar bag filled with nitrogen gas and the Tedlar bags were sealed. The sealed Tedlar bags were placed in a convection oven for 1 h at 25 °C or 80 °C. After 1 h, ammonia emission concentration of the foams over-coated by the deodorants was measured by an ammonia detector tube.

## 3. Results and Discussion

**Figure 2:**
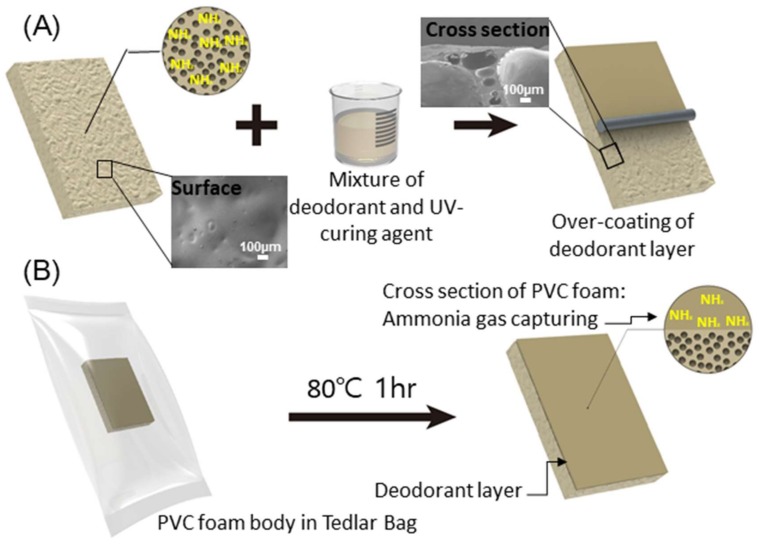
Schematics of experiments. (**A**) Over-coating process of deodorant layer on PVC foam. Inset: Scanning electron microscope (SEM) images of the surface and cross section of PVC foam before over-coating of the deodorant layer. (**B**) Ammonia emission/deodorization rate test using a Tedlar bag and the PVC foam after over-coating and absorption of ammonia gas by the deodorant layer.

Figure 2 shows the schematic illustration of the production process of eco-friendly polymer foam over-coated by a deodorant. In the forming process of PVC, the ammonia gas was captured due to the thermal decomposition of ADCA forming agent. For the stable and durable immobilization of deodorant materials on the polymer foam, the UV-curing agent was used as the bridging matrix (see detail in Experimental). A mixture of deodorant and UV-curing agent was over-coated on the PVC foam. To test the deodorization of time-releasing ammonia gas, the foam over-coated by the deodorant was sealed in the Tedlar bag filled with nitrogen gas and placed under 25 and 80 °C.

**Figure 3:**
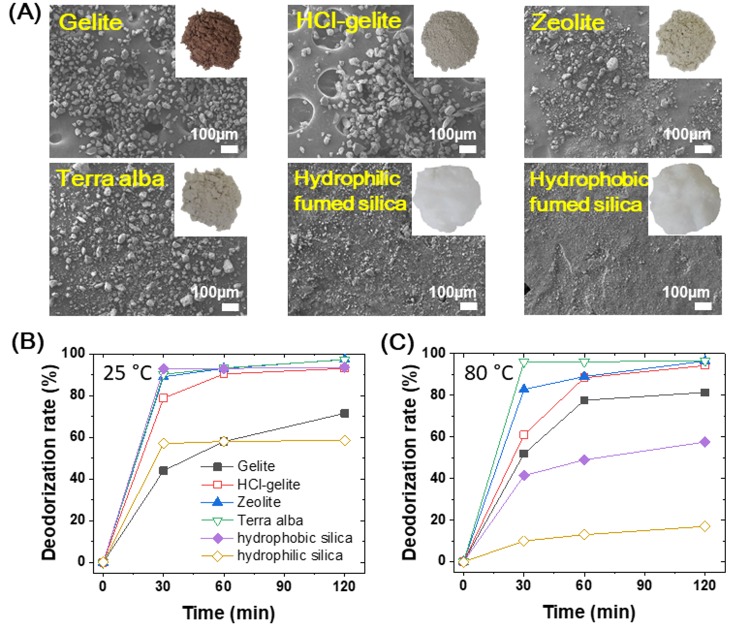
Deodorants and deodorization characteristics. (**A**) SEM images of original deodorants. Inset: photo images of original deodorant powders. (**B**,**C**) Ammonia deodorization rate of the original deodorants depending on the deodorization time at 25 °C (**B**) and 80 °C (**C**).

In this work, we used six different deodorants, gelite, HCl-gelite, zeolite, terra alba, and hydrophobic/hydrophilic fumed silica, which have been recognized by a good deodorizing ability [21,28,30]. Figure 3A shows the photo and SEM images of the original deodorizing materials. Hydrophobic fumed silica particles are a few micrometers, whereas the others have various sizes ranging from a few micrometers to tens of micrometers. A series of pozzolan is a natural deodorant due to the porous structure with a pore size of ~1 nm that is comparable to molecules such as ammonia [27]. Since the outer surface area of these porous materials is a few percent in the total surface area, most of active sites are on the inner surface. This feature means that for the molecular reaction the rate and the selectivity are mainly affected by the geometrical shape and the pore density. Accordingly, the deodorization rate (DR) for each of the deodorants can be changed by their characteristic morphologies and structures. However, because the brownish color of gelite has a disadvantage in direct use for the top layer of foam compared with other materials, the gelite was semi-permanently decolorized through the HCl treatment (HCl-gelite, Figure 3A). We examined the deodorization rate for the ammonia gas (ADR) with these six original materials (Figure 3B,C). The ADR was evaluated with an amount of 1 g of deodorant material. Figure 3B shows the ADR values as a function of time at room temperature (25 °C). The ADRs of terra alba, zeolite, HCl-gelite and hydrophilic fumed silica were drastically increased within 30 min and then saturated over 90% after 60 min. On the other hand, the gelite and hydrophobic fumed silica respectively showed an ADR value of 72% and 58%, respectively. Interestingly, the HCl-gelite showed a further enhanced deodorizing effect compared to the original gelite. Also, the hydrophobic fumed silica had a higher ADR value than the hydrophilic fumed silica. The former is presumably due to the ion exchanged surface of the gelite by a proton of HCl [35], where the protonated active sites can be easily exchanged from the proton to ammonia. In the latter, the hydrophobic surface caused by alkyl or polydimethylsiloxane chains may enhance the infiltration and absorption of ammonia [36]. Since the release rate of ammonia gas from the PVC foam is mostly dependent on the external temperature, it is also important to examine the ADR at high temperature. Figure 3C is for the ADR values of original materials measured at 80 °C. The ADR values that depend on the deodorizing time showed that the terra alba, zeolite, and decolorized gelite had a similar ability to deodorize the ammonia while both fumed silica had greatly decreased ADR values. This might be due to the short diffusing length and low thermal equilibrium temperature caused by relatively small particle size. On the other hand, for gelite it inversely increased up to ~80% within 1 h. This result made us not further consider the fumed silica as a deodorant of ammonia gas. Accordingly, we selected gelite, decolorized gelite, terra alba, and zeolite as the deodorants for further study.

**Figure 4:**
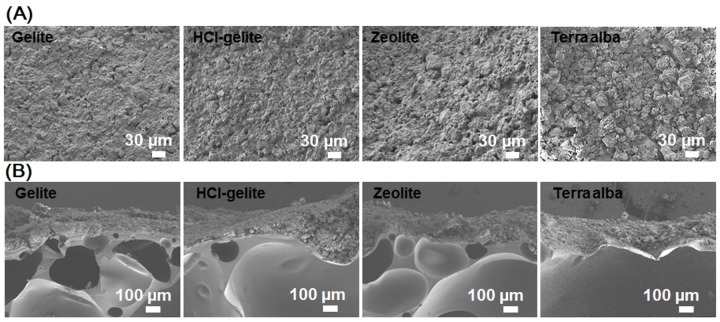
SEM images of the surface (**A**) and the cross section (**B**) of the complex foams after over-coated by the deodorant layers.

For the stable and durable over-coating layer, we mixed the deodorants with a UV-curing agent. The use of a UV-curing agent is helpful to avoid the sintering process that induces the decrease of specific surface area and porosity. Figure 4 shows SEM images of a UV-cured deodorant layer over the PVC foams. Figure 4A is for the top view and Figure 4B is for the cross section view of the complex PVC foams. From the top view (Figure 4A), it is hard to see an individual deodorant particle. This means that the UV-curing agent not only acted as a matrix to remarkably enhance the homogeneous packing and the bond between the microsized particles of deodorants, but also improved the bond between the deodorants and the PVC foam. However, it must be considered the ratio between the UV-curing agent and deodorants because of the active surface area of the deodorant particles. In the cross-section view (Figure 4B), the deodorants were coated with a film thickness of about 300 μm that is just 1% of the total foam thickness. In practice, the deodorization rate of ammonia gas releasing from the PVC foam can be controlled by the thickness of the deodorant layer. However, because the change of gravity of the final foam is also an important fact for further application, the deodorant layer thickness was appropriately adjusted as 1% of the total foam thickness. In the cross-section view, the sub-millimeter pores in PVC foam that must have taken a role of ammonia reservoirs are clearly shown.

**Figure 5:**
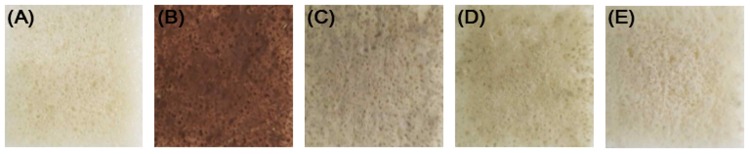
Photo images of the forms over-coated by the deodorants. (**A**) Reference (**B**) Gelite (**C**) HCl-gelite (**D**) Zeolite (**E**) Terra alba.

After over-coating the deodorants, the change of specific gravity and chromaticity of the PVC foams was measured and the results are shown in Figure 5 and listed on the Table 1. The photo images (Figure 5) show that the UV-curing agent did not affect the inherent color of the deodorants even after curing. The chromatic effect of the deodorant layer was qualitatively measured by the chromaticity experiment listed on the right of Table 1. In chromaticity, L* represents lightness and b* represents yellowness [37]. The L* values of complex foams with zeolite and terra alba were similar within 10% to that of the reference, whereas the L* of the complex foams using gelite and HCl-gelite relatively show a large difference, which was expected. The b* values, which are an important fact in chromaticity due to the practical preference, were changed relatively little in the foams using HCl-gelite and terra alba. In case of complex foams using gelite and zeolite, the change of b* value is more than 200% compared to the reference. The specific gravity of the complex foam increased slightly after coating, which must be due to the specific gravity of the coated deodorants.

**Figure 6:**
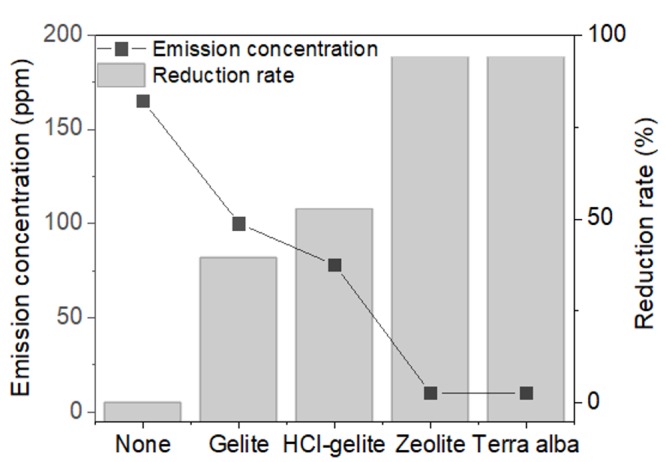
Relative ammonia reduction rate and emission concentration of the foams over-coated by the deodorants.

According to the process as schematically shown in Figure 2B, we tested the performance of ADR of a deodorant layer with complex foams. Figure 6 shows the comparison of relative ADR (rADR) of each of the complex foams corresponding to the deodorant materials. In all complex foams, the increment of rADR was observed, suggesting that the deodorant layers appropriately worked even after UV-cured on the PVC foams. When the gelite and HCl-gelite were used as a deodorant layer, the rADR was 40% and 53%, respectively. These values are lower than the ADRs of the raw materials, meaning that the UV-curing agent interrupted the diffusion and absorption of ammonia on the gelite and HCl-gelite. It is remarkable that zeolite and terra alba have a rADR value over 90%, which is similar to the ADR measured from the raw materials. Based on all results discussed above, it can be said that terra alba is the most suitable deodorant and then zeolite for the synthesis of eco-friendly foam. However, further study is needed to understand the cause of the different behaviors between gelite (HCl-gelite) and zeolite and terra alba in ADR performance.

## 4. Conclusions

In this work, we have developed deodorants of ammonia gas applying for the production of eco-friendly foams using nanoporous materials such as gelite, zeolite and terra alba. The deodorant layers over-coated on PVC foams worked well for the reduction of time-releasing ammonia. Based on the consideration of all results, although the deodorant layers using terra alba and zeolite showed a high ARD value over than 90%, the terra alba showed the best deodorizing performance of ammonia on the PVC foam. We believe that this work provides a promising solution to reduce the time-released hazardous gases from the synthetic polymer foams used in everyday life.

## Figures and Tables

**Table 1:** Specific gravity and chromaticity of the foams over-coated by the deodorants. L*: lightness, b*: yellowness.

Deodorant	Specific Gravity	Chromaticity	
		L*	B*
None (Referance)	0.160	52.73	4.66
Gelite	0.177 (1.03)	27.29	18.66
HCl-gelite	0.174 (0.91)	59.29	10.76
Zeolite	0.175 (0.92)	65.89	14.13
Terra alba	0.163 (0.80)	70.33	5.03

() Specific gravity of deodorant only.

## References

[1] Nielsen L.E., Landel R.F. (1994). Mechanical Properties of Polymers and Composites.

[2] Shastri V.P., Martin I., Langer R. (2000). Macroporous polymer foams by hydrocarbon templating. Proc. Natl. Acad. Sci. USA.

[3] Demir H., Sipahioğlu M., Balköse D., Ülkü S. (2008). Effect of additives on flexible PVC foam formation. J. Mater. Process. Technol..

[4] Verdu J., Zoller A., Marcilla A. (2013). Plastisol foaming process. Decomposition of the foaming agent, polymer behavior in the corresponding temperature range and resulting foam properties. Polym. Eng. Sci..

[5] Patterson J. (1998). Vinyl Foam: Effect of Density on Physical Properties. J. Vinyl Addit. Technol..

[6] Reglero Ruiz J.A., Vincent M., Agassant J.F., Sadik T., Pillon C., Carrot C. (2015). Polymer foaming with chemical blowing agents: Experiment and modeling. Polym. Eng. Sci..

[7] Heck R.L. (1998). A review of commercially used chemical foaming agents for thermoplastic foams. J. Vinyl Addit. Technol..

[8] Sivertsen K. (2007). Polymer Foams.

[9] Stehr J. (2016). Chemical blowing agents in the rubber industry. Past–present–and future?. Int. Polym. Sci. Technol..

[10] Hurnik H., Finzenhagen M., Jeblick W. (1987). New Blowing Agent Combination Based on Azodicarbonamide, Production Thereof and Use Thereof for Foaming Polymers. U.S. Patent.

[11] Sims G., Jaafar H. (1994). A chemical blowing agent system (CBAS) based on azodicarbonamide. J. Cell. Plast..

[12] Marshall R. (1991). Blowing agent decomposition in vinyl foams. J. Vinyl Technol..

[13] Naguib H.E., Park C.B., Panzer U., Reichelt N. (2002). Strategies for achieving ultra low-density polypropylene foams. Polym. Eng. Sci..

[14] Guo A., Javni I., Petrovic Z. (2000). Rigid polyurethane foams based on soybean oil. J. Appl. Polym. Sci..

[15] Radovanović R., Jašo V., Pilić B., Stoiljković D. (2014). Effect of PVC plastisol composition and processing conditions on foam expansion and tear strength. Hem. Ind..

[16] Lithner D., Larsson Å., Dave G. (2011). Environmental and health hazard ranking and assessment of plastic polymers based on chemical composition. Sci. Total Environ..

[17] Kim H.-Y. (2016). Worker Health Hazard and Risk Assessment of Formamide using in Workplaces in South Korea. J. Korean Inst. Gas.

[18] Slovak A. (1981). Occupational asthma caused by a plastics blowing agent, azodicarbonamide. Thorax.

[19] Hale R.C., La Guardia M.J., Harvey E., Mainor T.M. (2002). Potential role of fire retardant-treated polyurethane foam as a source of brominated diphenyl ethers to the US environment. Chemosphere.

[20] La Pagans E., Font X., Sánchez A. (2005). Biofiltration for ammonia removal from composting exhaust gases. Chem. Eng. J..

[21] Rahmani A., Mahvi A.H., Mesdaghinia A.R., Nasseri S. (2004). Investigation of ammonia removal from polluted waters by Clinoptilolite zeolite. Int. J. Environ. Sci. Technol..

[22] Zhang B.S., Lv X.F., Zhang Z.X., Liu Y., Kim J.K., Xin Z.X. (2010). Effect of carbon black content on microcellular structure and physical properties of chlorinated polyethylene rubber foams. Mater. Des..

[23] Kim B.G. (2008). Development of microwave foaming method for phenolic insulation foams. J. Mater. Process. Technol..

[24] Lee L.J., Zeng C., Cao X., Han X., Shen J., Xu G. (2005). Polymer nanocomposite foams. Compos. Sci. Technol..

[25] Gupta R., Kulkarni G.U. (2011). Removal of organic compounds from water by using a gold nanoparticle–poly (dimethylsiloxane) nanocomposite foam. ChemSusChem.

[26] Cao X., Lee L.J., Widya T., Macosko C. (2005). Polyurethane/clay nanocomposites foams: Processing, structure and properties. Polymer.

[27] Yokoi T. (2016). Characterization of Zeolites by Advanced SEM/STEM Techniques.

[28] Ackley M.W., Rege S.U., Saxena H. (2003). Application of natural zeolites in the purification and separation of gases. Microporous Mesoporous Mater..

[29] Villota R., Hawkes J.G., Cochrane H. (1986). Food applications and the toxicological and nutritional implications of amorphous silicon dioxide. Crit. Rev. Food Sci. Nutr..

[30] Kim S. (2009). The reduction of indoor air pollutant from wood-based composite by adding pozzolan for building materials. Constr. Build. Mater..

[31] Collins D.E., Richey F.A. (1992). Synthetic organic chemicals. Riegel’s Handbook of Industrial Chemistry.

[32] Jeong J., Kim T., Cho W.J., Chung I. (2013). Synthesis and decomposition performance of a polymeric foaming agent containing a sulfonyl hydrazide moiety. Polym. Int..

[33] Kim Y.S., Harris R., Davis R. (2012). Innovative Approach to Rapid Growth of Highly Clay-Filled Coatings on Porous Polyurethane Foam. ACS Macro Lett..

[34] Kim Y.S., Li Y.C., Pitts W.M., Werrel M., Davis R.D. (2014). Rapid Growing Clay Coatings to Reduce the Fire Threat of Furniture. ACS Appl. Mater. Interfaces.

[35] Lee J.F., Crum J.R., Boyd S.A. (1989). Enhanced retention of organic contaminants by soils exchanged with organic cations. Environ. Sci. Technol..

[36] Seredych M., Bandosz T.J. (2007). Mechanism of Ammonia Retention on Graphite Oxides:  Role of Surface Chemistry and Structure. J. Phys. Chem. C.

[37] MacAdam D.L. (1974). Uniform color scales. JOSA.

